# Psoralen Induced Liver Injury by Attenuating Liver Regenerative Capability

**DOI:** 10.3389/fphar.2018.01179

**Published:** 2018-10-22

**Authors:** Wang Zhou, Xi Chen, Guolin Zhao, Dengqiu Xu, Zhenzhou Jiang, Luyong Zhang, Tao Wang

**Affiliations:** ^1^Jiangsu Key Laboratory of Drug Screening, China Pharmaceutical University, Nanjing, China; ^2^Jiangsu Center for Pharmacodynamics Research and Evaluation, China Pharmaceutical University, Nanjing, China; ^3^Key Laboratory of Drug Quality Control and Pharmacovigilance, China Pharmaceutical University – Ministry of Education, Nanjing, China; ^4^State Key Laboratory of Natural Medicines, China Pharmaceutical University, Nanjing, China; ^5^Center for Drug Screening and Pharmacodynamics Evaluation, School of Pharmacy, Guangdong Pharmaceutical University, Guangzhou, China

**Keywords:** hepatotoxicity, psoralen, cycle arrest, liver regeneration, mTOR, mitochondrial damage

## Abstract

Psoralen is a major component of the common traditional Chinese medicine *Fructus Psoraleae* (FP). In this study, we focused on psoralen to explore FP-induced hepatotoxicity and the underlying mechanisms. The acute oral median lethal dose of psoralen in ICR mice was determined to be 1,673 mg/kg. C57BL/6 mice were administered psoralen intragastrically at doses of 400 mg/kg or 800 mg/kg, and were sacrificed 24 h after treatment. Changes in various hepatotoxicity indicators demonstrated that psoralen can cause mild liver injury in mice. Psoralen inhibited the viability of normal human liver L02 cells *in vitro* by inducing S-phase arrest. In addition, psoralen in both the mouse livers and L02 cells upregulated cyclin E1 and p27 protein levels. The 2/3 partial hepatectomy mouse model was used to further explore the effects of psoralen on the liver regeneration and hepatocellular cycle arrest *in vivo*. The results showed that the decrease of liver regenerative and self-healing capabilities induced by hepatocellular cycle arrest may play an important role in the hepatotoxicity of psoralen. The further mechanism researches indicated that psoralen-induced hepatotoxicity was associated with inhibition of mTOR signalling pathway and mitochondrial injury; furthermore, MHY, an mTOR activator, partly alleviated the inhibition of mTOR and S-phase cycle arrest induced by psoralen in L02 cells. In conclusion, in this study we showed for the first time, that psoralen significantly induced liver injury in mice; the decrease of liver regenerative and compensatory capabilities induced by hepatocellular cycle arrest may play an important role in the progression of hepatotoxicity associated with the upregulation of cyclin E1 and p27, as well as the inhibition of mTOR signalling and mitochondrial injury. Our findings may contribute to the reduction of hepatotoxicity risk induced by *Fructus Psoraleae.*

## Introduction

Psoralen is a major component of the common traditional Chinese medicinal plant *Fructus Psoraleae* (FP), the dried matured fruit of the leguminous plant *Psoralea corylifolia* L. that is widely used in Asian countries, particularly in China and India, for the treatment of osteoporosis, osteomalacia, arthralgia, asthma, vitiligo, and psoriasis (Cheung et al., 2009; Committee, 2015). However, based on several clinical reports, FP is associated with severe hepatotoxicity. For example, several patients experienced acute liver injury when only FP was used to treat vitiligo (Teschke and Bahre, 2009; Smith and MacDonald, 2014) or osteoporosis (Nam et al., 2005). Moreover, in the 9th, 16th, and 72nd Adverse Drug Reaction (ADR) Information Bulletins, the China Food and Drug Administration issued a warning against the potential hepatotoxicity of three types of FP-related preparations, including Baishi Wan (seven cases of liver injury in all eight ADR reports), ZhuangGuGuanJie Wan (47 cases of liver injury in all 158 ADR reports), and XianLingGuBao pills. In addition, in our previous study, the ethanol (EtOH) extract of FP was shown to induce hepatotoxicity in Sprague-Dawley rats; the chemical constituents of EtOH extract from FP were analysed using HPLC, and the results showed psoralen (21.7 mg/g) and isopsoralen (26.8 mg/g) were the two main components (Wang et al., 2012). Therefore, FP was considered a potential hepatotoxin. However, its hepatotoxic components and underlying mechanisms have not been well elucidated. Recently, investigated the hepatotoxicity of Bakuchiol, another major component of FP, and found that Bakuchiol only causes slight liver dysfunction in rats (Li et al., 2017). In another research focusing on the tissue distribution of coumarin components from FP, which contains 31.26% psoralen, among the six tissues analysed, the highest concentration of psoralen was found in the liver with extremely rapid distribution rate from blood into tissues and relatively slow elimination rate (Feng et al., 2010). In addition, psoralen and its isomer isopsoralen are used as quality control markers of FP according to criteria from the Pharmacopoeia of the People’s Republic of China (Committee, 2015). These data indicated that psoralen would be a relevant compound for further exploration of the hepatotoxicity associated with FP.

Several researches in China showed that treatment with high-dose FP power or its water extract only induced mild or slight liver injury in rats or mice, which indicates that the hepatotoxicity of FP is relatively low, or that the normal mouse and rat models cannot appropriately evaluate the hepatotoxicity of FP. The liver plays a central role in metabolic homeostasis; thus it is highly vulnerable to various toxins; and normal liver has remarkable regenerative capabilities allowing continuous performance of its functions despite the injuries. In addition, our unpublished results demonstrated that psoralen could induce normal human liver L02 cells cycle arrest. Based on these information, we speculate that the attenuation of liver regenerative and compensatory capabilities induced by hepatocellular cycle arrest play an important role in the hepatotoxicity of FP.

The 2/3 partial hepatectomy (PHx; also referred to as 2/3 PH or 70% PH) in rodents is a very common *in vivo* model for studying liver regeneration. In this model, the recovery of the lost liver mass and impaired liver function requires activation and proliferation of various cells and their interactions, including liver parenchymal cells and non-parenchymal cells. The hepatocellular proliferation is the most representative and important. Under normal conditions, most hepatocytes are mitotically quiescent (G0), yet these hepatocytes re-enter the cell cycle in response to tissue loss or injury. Cell cycle progression plays an indispensable role in tissue growth and regeneration of multicellular organisms. In the priming stage of liver regeneration induced by PHx, hepatic non-parenchymal cells must release various cytokines including growth factors such as HGF, as well as proinflammatory cytokines such as IL-6 and TNF-α, to promote entry of hepatocytes into the cell cycle from the quiescent phase (Michalopoulos and DeFrances, 1997; Fausto et al., 2006). Therefore, hepatocellular cycle arrest induced by any cause is likely to delay liver regeneration in PHx mice (Campbell et al., 2011; Lehmann et al., 2012). Based on the abovementioned considerations, we believe that the PHx mice model is appropriate for the investigation of liver regenerative capabilities and hepatocyte cell cycle arrest *in vivo*.

mTORC1 acts as a sensor of diverse intracellular and extracellular cues to adjust cellular biological processes appropriately, including cell proliferation, metabolism, and cell cycle progression (Laplante and Sabatini, 2012). AKT and AMPK are two key upstream regulators of mTORC1; AKT can indirectly activate mTORC1 (Ma et al., 2005); conversely, AMPK can inhibit mTORC1 (Gwinn et al., 2008). The typical downstream effectors of mTORC1 activation promote mRNA translation and protein synthesis by directly phosphorylating 4E-BP1 and p70S6K (Laplante and Sabatini, 2012). In recent years, numerous studies have focused on the role of AMPK-mTOR signalling pathway in cell cycle arrest. Many of these studies have shown that cell arrest in the G1/S phase of various human cells is associated with AMPK activation and mTOR inhibition (Rattan et al., 2005; Dalvai et al., 2010; Fogarty et al., 2016; Gwak et al., 2017).

Therefore, in this study, the hepatotoxicity of psoralen was evaluated and the possible toxic mechanisms involved *in vivo* and *in vitro* were explored, including cell cycle progression-relevant and mTOR signalling pathways.

## Materials and Methods

### Chemicals and Antibodies

Psoralen (purity > 98%, HPLC, Supplementary Figure 1) was purchased from Nanjing Jing Zhu Bio-Technology Co., Ltd. (Nanjing, China). Primary antibody to cyclin D1(2978), cyclin E1(20808), CDK1(77055), p27(3688), phospho-AMPKα(2535), AMPKα(2532), phospho-AKT(4060), phospho-mTOR(2971), mTOR(2983), phospho-4E-BP1(2855), 4E-BP1(9644), phospho-p70S6 kinase(9204), p70S6 kinase(2708) were purchased from Cell Signaling Technology (Danvers, MA, United States). Antibody to cyclin A2 (ab181591) was purchased from Abcam (Cambridge, MA, United States), AKT (sc-8312) and β-actin (sc-69879) were purchased from Santa Cruz Biotechnology (Dallas, TX, United States).

### Animals and Treatment

Six- to eight-week-old female ICR and C57BL/6 mice (weighing 18–22 g) were obtained from Shanghai Lingchang Biological Technology Co., Ltd. (Shanghai, China) and housed in a specific pathogen-free facility under a 12 h light–dark cycle with free access to water and food. All of the animals were kept in their cages for 1 week prior to experiments to allow acclimatisation to the laboratory conditions. All animal-related procedures were approved by the animal Ethics Council of China Pharmaceutical University.

The detailed process of measuring the acute oral LD50 is described in the following section, Determination of acute oral LD50. After the LD50 was determined, subsequent animal experiments for evaluating the hepatotoxicity of psoralen were performed, and C57BL/6 mice were randomly divided into three groups with eight mice per group. All of the mice were fasted (withholding of food but not water) overnight before dosing. The control group was treated intragastrically (IG) with 0.5% CMC-Na solution at a volume of 15 μL/g weight. The low- and high-dose groups were treated with the same volume of 400 mg/kg and 800 mg/kg psoralen, respectively. After 24 h, mice were sacrificed, and blood was collected without anticoagulants to obtain serum. Several organs were isolated and weighed including the liver, kidney, lung, and gallbladder. Next, the livers were fixed in 10% neutral formalin or frozen in liquid nitrogen.

In the liver regeneration experiment, C57BL/6 mice were subjected to PHx according to the method of Mitchell and Willenbring (2008). Anaesthesia was induced by isoflurane inhalation (2–4%) using the anaesthetic gas machine. The livers were resected and weighed at different times. Mice were divided into a control group and PH group. With the exception of the control group, all of the mice in the PH group were subjected to PHx. The PH group was further divided into four groups based on the time of sacrifice after hepatectomy: 12 h, 24 h, 48 h, and 72 h groups. In addition, each PH group was subdivided into two groups: vehicle group (treated with 0.5% CMC-Na) and psoralen group (treated with psoralen 200 mg/kg). In all of the PH groups, mice were pretreated with CMC-Na or psoralen for 24 h and on the second day, were dosed once again prior to hepatectomy, which was performed immediately thereafter. Mice were sacrificed 12 h or 24 h after hepatectomy (12 or 24 h group, respectively), and mice in the control group were sacrificed at the same time as the mice in the 12 h group. In the 48 and 72 h groups, mice were dosed once a day after hepatectomy and were sacrificed 48 and 72 h after hepatectomy, respectively. The liver and body weights were recorded at the time of animal sacrifice and were used to calculate the liver regeneration rate. Liver regeneration rate = (W1 − [W0 − W2])/W0 × 100%, where W0 is the gross weight of liver before resection, an estimated result based on the weight of the resected liver divided by 0.7; W1 is the weight of the liver after PHx when the mice were sacrificed; and W2 is the weight of the resected liver after mice are subjected to PHx surgery.

### Determination of Acute Oral LD50

The acute oral median lethal dose (LD50) was determined using the limit dose test of Up and Down Procedure (UDP) according to the OECD/OCDE GUIDELINES FOR THE TESTING OF CHEMICALS (OCDE, 2008). The treatment schedule and psoralen doses were designed and succedent estimation of LD50 using a computer software, the Acute Oral Toxicity (Guideline 425) Statistical Program (AOT425StatPgm, version 1.0). Based on our previous relevant research (Zhao et al., 2017), the starting dose was selected at 350 mg/kg body weight and the limit dose at 2,000 mg/kg, Sigma at 0.3.

### Cell Culture and Viability Assay

The human hepatocyte cell line L02 was obtained from the China Cell Culture Center (Shanghai, China), and the hepatoma cell line HepG2 was obtained from the American Type Culture Collection (Manassas, VA, United States). Cells were cultured in Dulbecco’s Modified Eagle Medium high-glucose medium (L02) or Minimum Essential Medium (HepG2) supplemented with 10% foetal bovine serum (Gibco, Waltham, MA, United States), 100 U/mL penicillin, and 100 U/mL streptomycin at 37°C in a humidified atmosphere with 5% CO_2_. In addition, 1 mM sodium pyruvate was added to the medium for culturing HepG2 cells. Morphological changes and cell survival were monitored under an inverted phase-contrast microscope (Olympus, Tokyo, Japan). Cell viability was measured using the 3-(4,5-dimethylthiazol-2yl)-2,5-diphenyltetrazolium bromide (MTT) assay. In addition, every 3 or 6 h in a 48 h period, cell viability and total protein (TP) were measured to monitor cell proliferation and analyse cycle-related protein levels. In this study, 0 h was defined as the time when the cells were just beginning to completely adhere, which was 6 h after cell seeding.

### Serum Biochemical Analysis

Serum alanine transaminase (ALT), aspartate transaminase (AST), alkaline phosphatase (ALP), total bile acids (TBAs), and total bilirubin (TBIL) were determined using an HITACHI7080 Automatic Clinical Analyzer (HITACHI, Tokyo, Japan). Serum albumin (ALB) and TP, which reflect the changes in liver anabolism, were analysed using a commercial kit according to the manufacturer’s protocol (Whitman Biotech, Nanjing, China).

### Histologic Examination

The livers were isolated and fixed in 10% neutral-buffered formalin, and then were embedded in paraffin, sectioned and stained with haematoxylin and eosin (HE). Microscopic observation and histopathological assessment were performed by a professional pathologist (Wenxia Bai, Jiangsu Provincial Medicine Institute, Nanjing, China). In addition, the paraffin-embedded liver sections were also used for immunohistochemical staining. The primary antibodies of anti-PCNA (MAB-0145) and anti-Ki67 (GT210104) were obtained from MXB Biotechnologies (Fujian, China) and Gene Tech (Shanghai, China), respectively. Diaminobenzidine (DAB) was applied as the chromogen, which resulted in a brown reaction product and counterstained with hematoxylin. Both the number of PCNA-positive and Ki67-positive hepatocytes in ten random high-power fields were counted using Image Pro Plus 6.0 software.

### Lactate Dehydrogenase (LDH) Leakage Assay and Determination of ATP Content

Lactate dehydrogenase (LDH)-release assay was used to detect the cytotoxicity of psoralen. Cells were seeded into 24-well plates, and then treated with DMSO or psoralen. 10X lysis solution was used to generate a maximum LDH release as the positive control wells. LDH-leakage level was measured via transferring 50 μL culture supernatants from all of the wells to a fresh 96-well plate and the subsequent processes were performed according to the instructions of CytoTox 96^®^ Non-Radioactive Cytotoxicity Assay Kit (Promega, Madison, WI, United States). The percent cytotoxicity = 100 × Experimental LDH Release (OD490)/Maximum LDH Release (OD490). In addition, the cells at the bottom of 24-well plate were used to detect the intracellular ATP content according to the manufacturer’s protocol of CellTiter-Glo^®^ 2.0 Assay Kit (Promega). The ATP content was normalised against the cellular TP content, which was quantified using the BCA Protein Assay Kit (Beyotime, Nanjing, China). The mitochondria of liver tissues were isolated using the Tissue Mitochondria Isolation Kit (Beyotime), and the ATP and protein content were measured using the same method as above.

### Apoptosis and Cell Cycle Analysis

Cell Apoptosis was quantified using a FITC Annexin V Apoptosis Detection Kit (BD Bioscience, San Jose, CA, United States). After treatment, the cells were harvested and resuspended in 100 μL buffer solution with 5 μL Annexin V-FITC and 5 μL propidium iodide (PI). The stained cells were analysed using BD FACSCalibur flow cytometer (Becton–Dickinson, Franklin Lakes, NJ, United States). In cell cycle experiment, after treatment, the cells were harvested and fixed in 70% alcohol for 12 h, and then washed twice with PBS and stained with PI using a Cell Cycle and Apoptosis Analysis Kit (Beyotime). The cell cycle distribution was analysed using BD FACSCalibur flow cytometer (Becton–Dickinson).

### Mitochondria Labelling

MitoTracker^®^ Red CMXRos (Invitrogen, Waltham, MA, United States), a cell-permeant fluorescent probe, was used to label mitochondria. After treatment, the cells were rinsed twice with PBS and labelled mitochondria using MitoTracker^®^ Red CMXRos, and then fixed with 4% paraformaldehyde. DAPI was utilised for nuclear staining. Imaging observation of mitochondria was performed in laser scanning confocal microscopy (FV1000, Olympus, Tokyo, Japan).

### Transmission Electron Microscopy

After the mice were sacrificed, liver tissues were isolated immediately and then trimmed to less than 1 mm^3^, and fixed in 2.5% neutral-buffered glutaraldehyde at 4°C. The follow-up process and preparation of ultrathin sections were performed by Nanjing Medical University Analysis Center (Nanjing, China). The stained samples were examined using a JEM-1010 electron microscope (JEOL, Peabody, MA, United States).

### RNA Isolation and Real-Time Quantitative PCR

Total RNA was extracted from liver tissues using Trizol method according to the manufacturer’s protocol (Vazyme Biotech, Nanjing, China) and converted to cDNA using the cDNA synthesis kit HiScript^®^Q RT SuperMix for qPCR (Vazyme Biotech). Real-time qPCR was performed in Applied Biosystems System using SYBR^®^ Green Master Mix (Vazyme Biotech). The thermal cycler conditions were as follows: 5 min at 95°C, followed by 40 cycles of 10s at 95°C and 30 s at 60°C. A melting curve analysis was carried out for each reaction from 60 to 95°C. mRNA expression levels were evaluated using ΔΔCT method and normalised to β-actin. All primer sequences were shown in Supplementary Table 1.

### Western Blot

Total proteins were extracted using RIPA Lysis Buffer supplemented with phosphatase and protease inhibitors (Beyotime). Proteins concentration was quantified using BCA Protein Assay Kit (Beyotime). The protein samples were separated using sodium dodecyl sulphate polyacrylamide gel electrophoresis in 6–12%, and then transferred to a nitrocellulose membrane (Millipore, Billerica, MA, United States). After blocking, the membranes were incubated with primary antibodies at 4°C overnight under shaking conditions. Protein bands were visualised using Tanon 4200 Chemiluminescent Imaging System (Shanghai, China). The relative density of immunoreactive bands was analysed using ImageJ software and β-actin as a loading control. The striping buffer was obtained from Beyotime.

### Statistical Analysis

Data presented in the form of bar graphs are means ± standard deviations (SDs). Statistical analyses between two groups were performed using the Student’s two-tailed *t*-test. Comparisons among three or more groups were performed using one-way analysis of variance with Tukey’s *post hoc* test. Statistics were performed using PASW Statistics 18, and *p* < 0.05 was considered statistically significant.

## Results

### Determination of Acute Oral LD50 in Mice

The toxic dose of psoralen has not been determined *in vivo*; thus, we used the up-and-down procedure to estimate the LD50 (Supplementary Tables 1,2). The mortality and survival times associated with each dose are shown in Table 1, and the changes in body weight are presented in Supplementary Figures 1–4. The oral LD50 of psoralen in mice was determined to be 1,673 mg/kg body weight.

**Table 1:** Mortality induced by gavage administration of psoralen to ICR mice and survival times corresponding with each treatment.

Dose (mg/kg)	Mortality	Survival times
350	0/1	>14 days
700	0/1	>14 days
1,390	1/3	23 h
2,000	2/3	44 h, 20 h

### Psoralen Induced Hepatotoxicity in Mice

**FIGURE 1:**
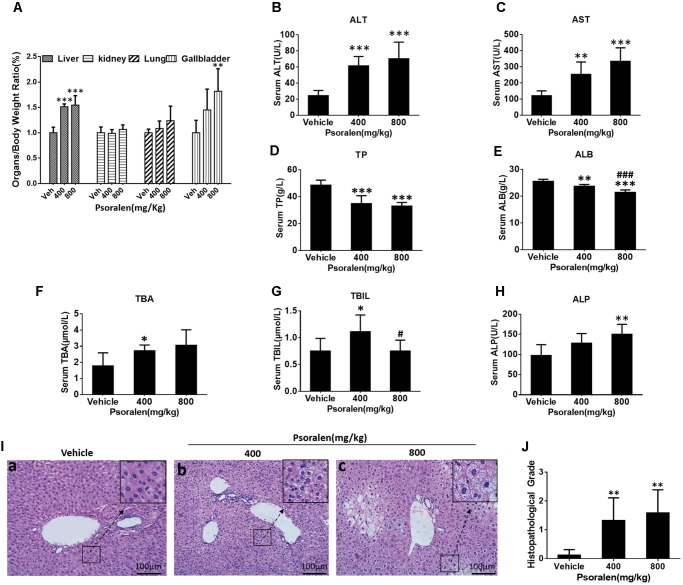
Treatment with psoralen induced liver injury *in vivo*. C57BL/6 mice were treated with 0.5% CMC-Na solution (vehicle group), 400 mg/kg, or 800 mg/kg of psoralen for 24 h. **(A)** Several organ-to-body weight ratios. **(B)** Serum ALT, **(C)** AST, **(D)** TP, **(E)** ALB, **(F)** TBA, **(G)** TBIL, **(H)** ALP levels. **(I)** Liver sections were stained with haematoxylin and eosin (HE), the representative micrographs of vehicle, 400 mg/kg, and 800 mg/kg psoralen groups are shown in **(a–c),** respectively; original magnification, 200×. **(J)** The overall degree of liver injury in each group was expressed as histopathological grade. Values are means ± SDs (*n* = 8). ^∗^*p* < 0.05, ^∗∗^*p* < 0.01, ^∗∗∗^*p* < 0.001 versus control group. ^#^*p* < 0.05, ^###^*p* < 0.001 versus 400 mg group.

To determine the liver injury induced by psoralen *in vivo*, additional animal experiments were performed. The results indicated that the liver/body weight ratio (Figure 1A) as well as serum alanine aminotransferase (ALT; Figure 1B) and AST (Figure 1C) levels significantly increased, and serum TP (Figure 1D) and ALB (Figure 1E) levels significantly decreased in a dose-dependent manner 24 h after psoralen administration. In addition, the gallbladder/body weight ratio (Figure 1A), serum TBA (Figure 1F), TBIL (Figure 1G), and ALP (Figure 1H) were slightly elevated in the psoralen group. Liver HE staining results showed diffuse hepatocellular oedema and even ballooning in local areas after psoralen treatment (Figure 1I), particularly in the high-dose group after psoralen administration (Figure 1I-c). Semi-quantitative analyses were used to evaluate the degree of injury (Figure 1J). These results showed that psoralen could induce liver injury in mice.

### Psoralen Inhibited L02 Cell Proliferation by Inducing S-Phase Arrest

**FIGURE 2:**
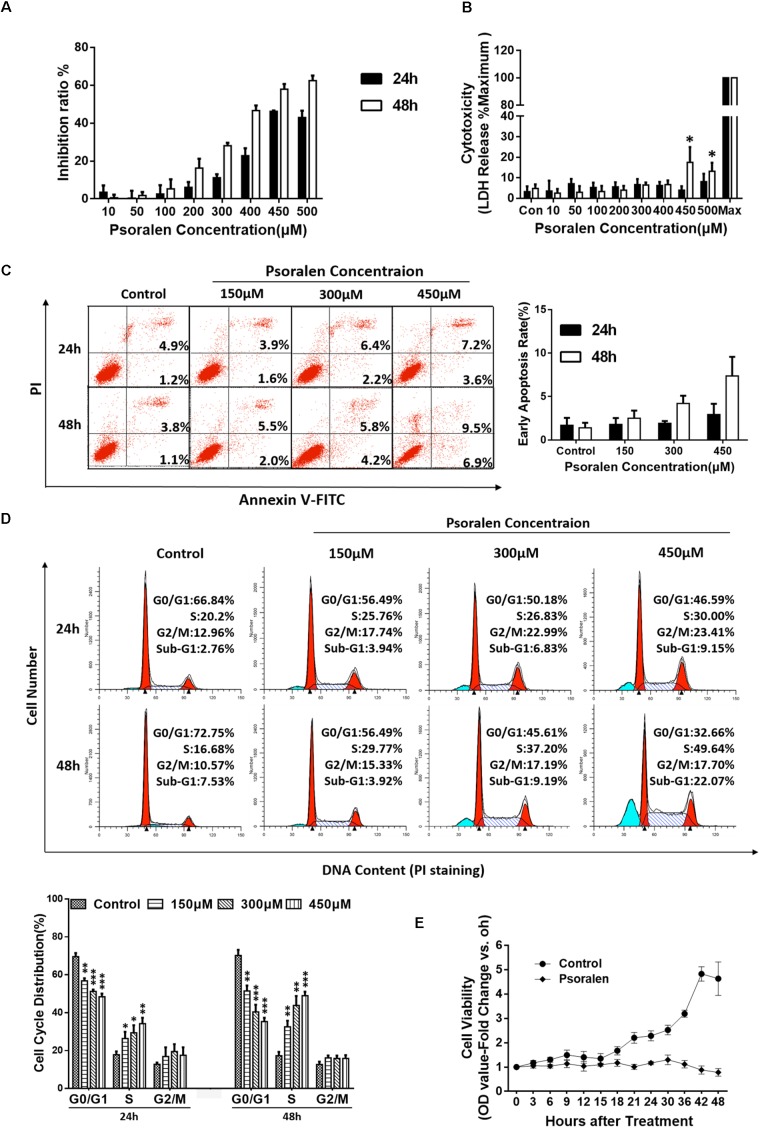
Psoralen inhibited the viability of L02 cells by inducing S-phase arrest. L02 cells were treated with DMSO or psoralen. **(A)** MTT assay was used to determine L02 cell viability. **(B)** LDH release assay was used to evaluate the cell membrane integrity. **(C)** Apoptotic analyses were performed using Annexin V-FITC/PI double staining. Left panel: representative results of flow cytometry analysis; right panel: quantitative statistics of early apoptotic cells. **(D)** Cell cycle analysis using PI staining. Upper panel: representative results of flow cytometry analysis; lower panel: quantitative statistics of cell cycle distribution. **(E)** MTT assay was used to monitor cell proliferation. All experiments were performed three times independently and the quantitative data are shown as the means ± SDs. ^∗^*p* < 0.05, ^∗∗^*p* < 0.01, ^∗∗∗^*p* < 0.001 versus control group.

To explore the mechanisms underlying psoralen-induced hepatotoxicity, two common cell lines for hepatocyte-related research, HepG2 and L02, were selected for preliminary *in vitro* studies. The results showed that psoralen suppressed the viability of L02 cells (Figure 2A) and HepG2 cells (Supplementary Figure 3A) in concentration- and time-dependent manners, whereas the LDH assay showed that little LDH was released under the same conditions (Figure 2B and Supplementary Figure 3B). In L02 cells, extracellular LDH levels did not significantly change at the 400 μM psoralen concentration. However, at 450 μM, the release of LDH slightly increased (approximately 10% higher than in the control well); 400 μM and 450 μM psoralen inhibited 50–60% of cell viability (Figures 2A,B). The MTT and LDH assay results indicated that psoralen inhibited the viability of L02 and HepG2 cells mainly by suppressing cell proliferation rather than causing cell death.

To verify this hypothesis, additional experiments were performed. Apoptosis analysis showed no obvious apoptotic events in L02 (Figure 2C) and HepG2 cells (Supplementary Figure 3C) despite the slight increase in statistical significance after psoralen treatment. However, in cell cycle analysis, psoralen induced significant S-phase arrest in L02 cells in time- and dose-dependent manners (Figure 2D), but no statistically significant change in the cycle distribution of HepG2 cells was observed (Supplementary Figure 3D).

In addition, the cell viability assay used to monitor cell proliferation by successive measures every 3 and 6 h in a 48 h period showed results consistent with our hypothesis; the cell viability of L02 cells in the control wells gradually increased with the increase in time and the growth curve was close to the typical “S” shape, but the viability remained nearly unchanged after treatment with 450 μM psoralen, indicating that L02 cells were just not proliferating rather than dying. At the 42 and 48 h time points, cell viability slightly decreased (Figure 2E), similar to the increased LDH release observed in cells treated with 450 μM psoralen for 48 h (Figure 2B). In general, the abovementioned results indicated that psoralen inhibited the growth and proliferation of L02 cells mainly by inducing S-phase arrest instead of causing cell apoptosis or death. However, we were unable to determine why the cell viability of HepG2 cells was inhibited. Thus, only L02 cells were used for subsequent research on molecular mechanisms.

### Psoralen Regulated the Expression of Cycle Progression-Relevant Proteins *in vivo* and *in vitro*

**FIGURE 3:**
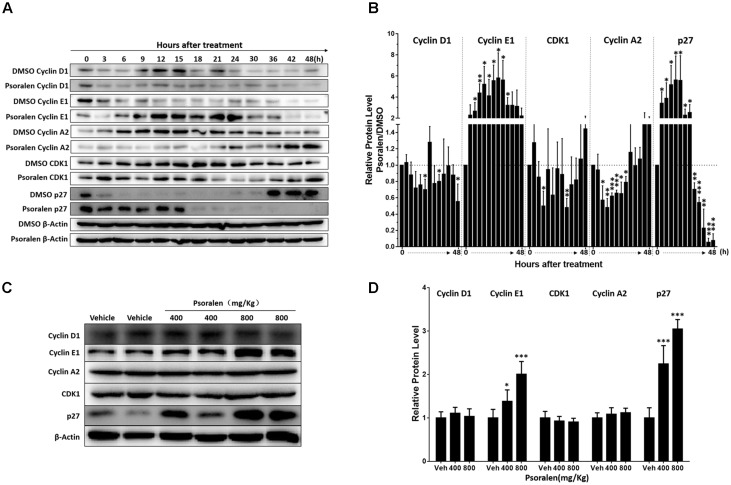
Changes in cell cycle progression-related proteins after psoralen treatment in L02 cells and mice. L02 cells were treated with DMSO or psoralen and incubated for various time periods. When the cells were just beginning to completely adhere (defined as 0 h), the total protein was collected successively every 3 and 6 h during a 48 h period. All experiments were performed three times independently. **(A)** Representative immunoblots for the protein levels of cyclin D1, cyclin E1, cyclin A2, CDK1, and p27. **(B)** Relative protein levels of cyclin D1, cyclin E1, cyclin A2, CDK1, and p27. The density of immunoreactive bands was quantified using ImageJ software. Density values were normalised to β-actin and then expressed as the relative expression levels between DMSO-treated and psoralen-treated. C57BL/6 mice were treated with 0.5% CMC-Na solution (vehicle group), or 400 mg/kg or 800 mg/kg psoralen for 24 h. **(C)** Representative immunoblots for the protein levels of cyclin D1, cyclin E1, cyclin A2, CDK1, and p27. **(D)** The correspondingly relative protein levels were quantified by scanning band intensity and normalised to β-actin (*n* = 6). Data are means ± SDs (*n* = 6). ^∗^*p* < 0.05, ^∗∗^*p* < 0.01, ^∗∗∗^*p* < 0.001 versus DMSO group or vehicle group.

Based on the abovementioned results, the potential molecular mechanisms of psoralen-induced S-phase arrest were analysed using Western blotting to detect the expression levels of several G1/S phase progression-relevant proteins. Combined with current theories on the regulation patterns of multiple proteins associated with cell cycle progression and our results showing the change in S phase-related protein levels in the solvent (DMSO) control group, we determined that most L02 cells entered the S phase approximately 6–9 h after cell adhesion when cyclin E1 was rapidly degraded and cyclin A2 levels rapidly increased with higher CDK1 levels, and cyclin D1 slightly increased over time or at least remained at stable levels (Figure 3A). Furthermore, rapid reduction in p27 levels was determined only 3 h after adhesion and remained extremely low until 36 h when the cell confluency was approximately 85–90%. Contrary to the control group, psoralen treatment led to rapidly and significantly elevated cyclin E1 levels at 3 h after adhesion until 24 h, accompanied by suppression of elevated cyclin A2 levels despite a slight increase at 42 and 48 h. The p27 levels remained higher until 15 h after adhesion and cyclin D1 levels slightly decreased (Figure 3A). The relative expression levels of these cycle-related proteins in control and psoralen groups at each time point are shown in Figure 3B. Based on these results, psoralen-induced S-phase arrest in L02 cells was mainly associated with the upregulation of cyclin E1 and p27, as well as downregulation of cyclin A2.

In addition, similar changes in these proteins were detected in the liver of mice. Psoralen dose dependently increased hepatic cyclin E1 and p27 protein levels in mice, whereas unlike in L02 cells, cyclin A2 protein levels were not significantly changed in the liver (Figures 3C,D). There was no significant difference in the protein level of hepatic cyclin D1 and CDK1 between the vehicle group and psoralen group (Figures 3C,D).

### Psoralen Toxicity Increased in PHx Mice

**FIGURE 4:**
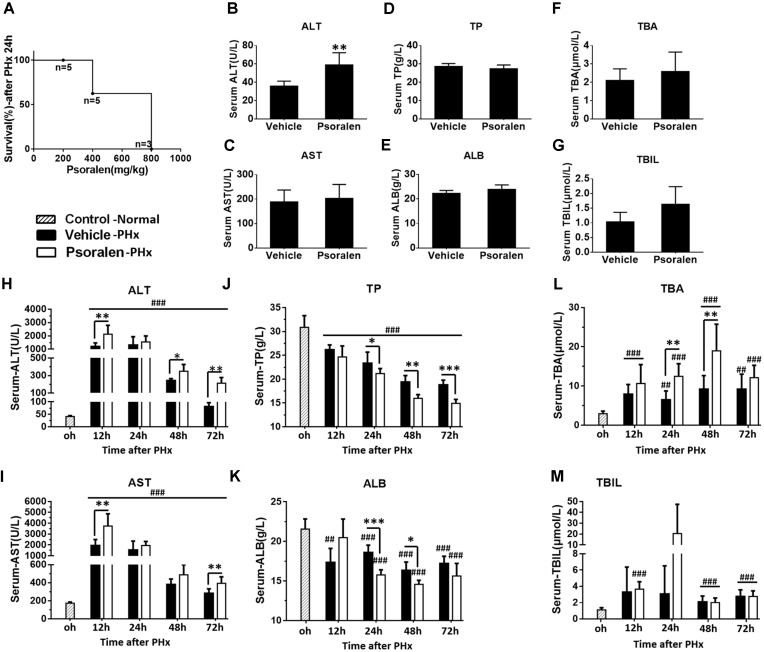
Psoralen toxicity increased and psoralen aggravated acute liver injury in PHx mice. C57BL/6 mice were subjected to PHx. **(A)** Mortality of PHx mice after 200 mg/kg, 400 mg/kg, or 800 mg/kg psoralen treatment. **(B–G)** Normal C57BL/6 mice were treated with 200 mg/kg of psoralen or 0.5% CMC-Na for 48 h (*n* = 8). **(B)** Serum ALT, **(C)** AST, **(D)** TP, **(E)** ALB, **(F)** TBA, **(G)** TBIL levels were determined. **(H–M)** C57BL/6 mice were subjected to PHx and treated with 200 mg/kg of psoralen or 0.5% CMC-Na. **(H)** Serum ALT, **(I)** AST, **(J)** TP, **(K)** ALB, **(L)** TBA, **(M)** TBIL levels were determined. Data are means ± SDs (*n* = 6–8). ^∗^*p* < 0.05, ^∗∗^*p* < 0.01, ^∗∗∗^*p* < 0.001 versus vehicle group. ^##^*p* < 0.01, ^###^*p* < 0.001 versus control group.

To determine the effects of psoralen on liver regeneration and hepatocyte cell cycle arrest *in vivo*, PHx was performed in mice. Whether mice can tolerate 800 mg/kg psoralen after PHx was uncertain; thus, a small-scale test was performed to estimate the safe dose of psoralen in this model. Unexpectedly, the mice receiving 800 mg/kg psoralen all died within 24 h after PHx. Moreover, the mortality rate of five mice treated with 400 mg/kg of psoralen was 40%. When treated with 200 mg/kg psoralen, all of the mice survived; however, these mice required more time to awaken from the anaesthetic state than those treatment with CMC-Na. The mortality rate of PHx mice after psoralen treatment is shown in Figure 4A; the results showed that 70% resection of the liver mass caused an increase in psoralen toxicity.

### Psoralen Aggravated Acute Liver Injury in PHx Mice

Subsequently, 200 mg/kg psoralen was selected for further research. To further determine whether 200 mg/kg psoralen could induce hepatotoxicity, C57BL/6 mice were treated with psoralen or vehicle for 48 h, because in the PHx model, multiple indices were most significantly altered at 24–48 h after PHx. The results showed that only serum ALT mildly increased (Figure 4B) and the other serum markers of hepatotoxicity were not significantly different between the two groups (Figures 4C–G).

In PHx mice, serum biochemical analysis indicated that hepatectomy led to severe acute liver injury, which was aggravated by psoralen. The results showed that serum ALT and AST levels significantly increased to a peak at 12 h after PHx and then gradually decreased. Compared to the vehicle group, ALT and AST levels were significantly increased in the psoralen group and decreased more slowly (Figures 4H,I). Serum TP and ALB levels were significantly decreased after PHx and were significantly lower in the psoralen group than in the vehicle group at 24, 48, and/or 72 h after PHx (Figures 4J,K). Serum TBA levels gradually increased after PHx, but in the psoralen group, TBA levels were significantly higher at 24 and 48 h after PHx (Figure 4L). Serum TBIL levels slightly increased after PHx; however, no significant differences were observed between the vehicle and psoralen groups (Figure 4M).

### Psoralen Delayed Liver Regeneration Associated With Hepatocellular Cycle Arrest in PHx Mice

**FIGURE 5:**
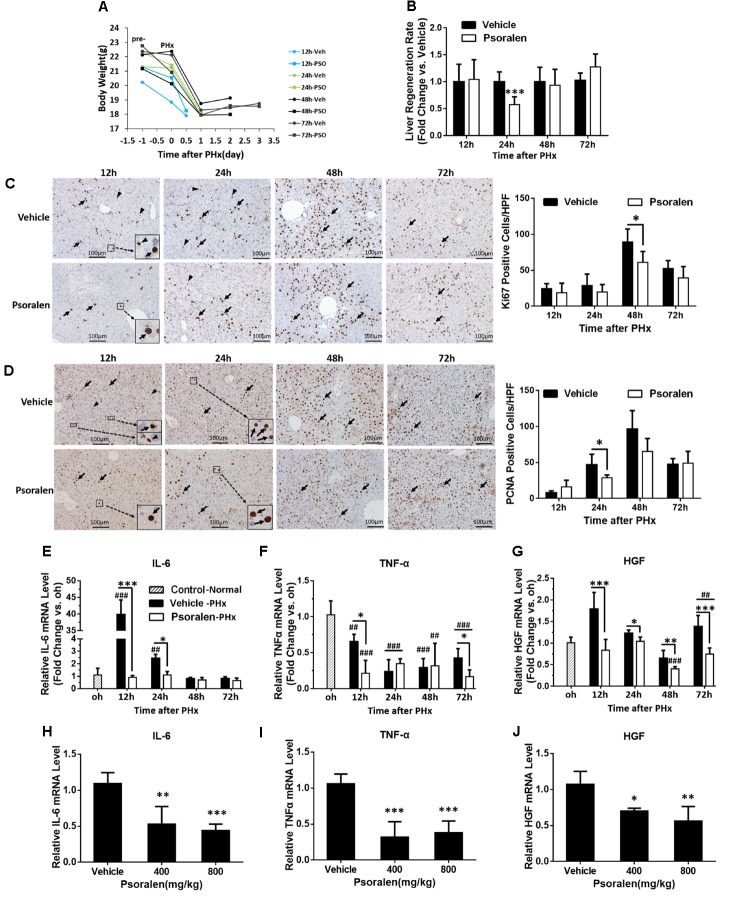
Psoralen delayed liver regeneration in PHx mice. C57BL/6 mice were subjected to PHx and treated with 200 mg/kg of psoralen or 0.5% CMC-Na. **(A)** Changes in body weight over time in PHx mice treated with CMC-Na or psoralen. **(B)** Relative liver regeneration ratio in PHx mice treated with CMC-Na or psoralen. **(C,D)** Liver sections for the evaluation of hepatocyte proliferation with anti-Ki67 **(C)** and anti-PCNA **(D)** staining, original magnification, 200×. Left panel: representative immunohistochemical images, proliferative hepatocytes (black arrow) or inflammatory cells (black arrowhead) are shown; right panel: number of Ki67- or PCNA-positive hepatocytes/high-power field (HPF). Ten random HPF views were counted in liver sections of each mouse. **(E–G)** Hepatic mRNA expression levels of IL-6 **(E)**, TNF-α **(F)**, and HGF **(G)** in PHx mice were determined using real-time qPCR. The relative mRNA levels were normalised to control mice (control group); β-actin was used as a reference gene. **(H–J)** Hepatic mRNA expression levels of IL-6 **(H)**, TNF-α **(I)**, and HGF **(J)** of normal mice treated with 400 mg/kg or 800 mg/kg of psoralen or CMC-Na. Data are means ± SDs (*n* = 6–8). ^∗^*p* < 0.05, ^∗∗^*p* < 0.01, ^∗∗∗^*p* < 0.001 versus the vehicle group; ^##^*p* < 0.01, ^###^*p* < 0.001 versus control group.

The results showed that body weight slightly decreased in most of the mice after pretreatment, followed by a greater decrease after PHx (Figure 5A). The liver regeneration ratio in the psoralen-treated group was significantly lower than that in the vehicle-treated group at 24 h after PHx, and then the ratio in the psoralen-treated group slightly increased but without statistical significance (Figure 5B).

During liver regeneration, hepatocellular proliferation is very important for restoring the lost liver mass and impaired liver function. To evaluate hepatocyte proliferation, immunohistochemistry staining was used to assess the expression of two principal markers: Ki-67, which is present during all active phases of the cell cycle (Bruno and Darzynkiewicz, 1992); and PCNA, the S-phase marker expressed in the DNA synthesis phase (Leonardi et al., 1992). The results showed that the number of Ki-67-positive cells gradually increased after PHx and peaked at 48 h in both groups; however, in the psoralen group, the peak value was significantly lower than that in the vehicle group (Figure 5C). Similarly, the number of PCNA-positive cells gradually increased after PHx and peaked at 48 h in both groups; the peak value in the psoralen group was also lower than that in the vehicle group, but not significantly (Figure 5D). Significantly fewer PCNA-positive cells were observed in the psoralen group than in the vehicle group at 24 h after PHx (Figure 5D). However, many PCNA-positive cells, which were smaller than hepatocytes and mostly not round, were observed in the Ki-67 immunohistochemistry staining in the vehicle group at 12 and 24 h after PHx (Figure 5C), and in PCNA immunohistochemistry staining at 12 h after PHx (Figure 5D). However, these types of cells were rarely observed in the psoralen group (Figures 5C,D), and as such were initially considered inflammatory cells. In addition, subsequent mRNA expression analysis of IL-6 and TNF-α supported this viewpoint.

In the priming stage of liver regeneration, several cytokines played a critical role including IL-6, TNF-α, and HGF. PCR results showed that hepatic IL-6 mRNA levels were significantly elevated compared to the control and vehicle groups at 12 and 24 h after PHx (Figure 5E). Because TNF-α mRNA levels were decreased in both vehicle and psoralen groups compared to the control group, the increase in TNF-α mRNA levels may have occurred earlier after PHx (Yin et al., 2011); however, psoralen treatment induced lower mRNA levels than vehicle treatment at 12 and 72 h after PHx (Figure 5F). In addition, HGF mRNA levels were decreased in the psoralen-treated group at all four time points after PHx compared to the vehicle group (Figure 5G). Similar results were observed in normal mice; psoralen dose-dependently induced a reduction in IL-6, TNF-α, and HGF mRNA expression levels (Figures 5H–J).

**FIGURE 6:**
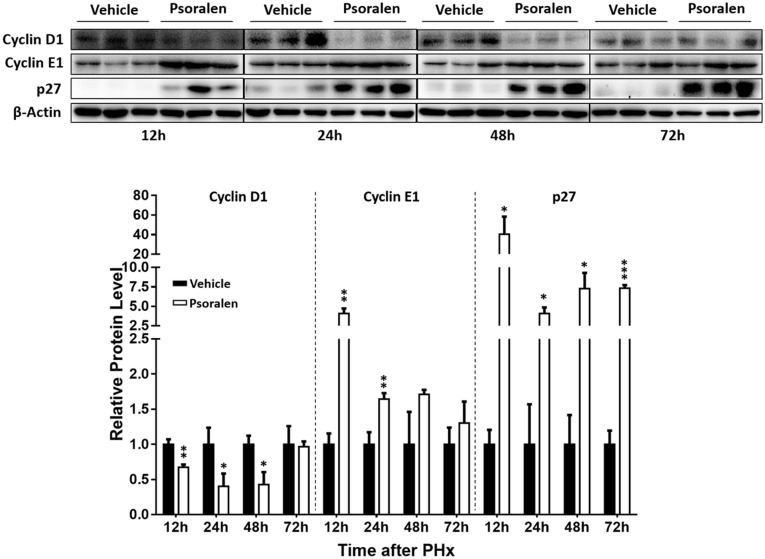
Psoralen decreased cyclin D1 and increased cyclin E1 and p27 protein levels in PHx mice. C57BL/6 mice were subjected to PHx and treated with 200 mg/kg of psoralen or 0.5% CMC-Na. Upper panel: representative immunoblots for the protein levels of cyclin D1, cyclin E1, and p27; lower panel: the relative protein levels were quantified and normalised to β-actin. Data are means ± SDs (*n* = 6). ^∗^*p* < 0.05, ^∗∗^*p* < 0.01, ^∗∗∗^*p* < 0.001 versus vehicle group.

Furthermore, Western blot analysis showed that p27 protein levels significantly increased in the psoralen group at all four time points after PHx (Figure 6), and psoralen significantly increased cyclin E1 protein levels at 12 and 24 h after PHx (Figure 6), similar to previous results in normal mice (Figures 3C,D). However, psoralen significantly inhibited cyclin D1 protein expression at 12, 24, and 48 h after PHx (Figure 6), which indicates that arrest of both the S phase and G1 phase was induced by psoralen in PHx mice. Collectively, these results indicate that psoralen significantly delayed liver regeneration associated with hepatocellular cycle arrest by downregulating cyclin D1 levels and upregulating cyclin E1 and p27 levels.

### Psoralen-Induced Inhibition of the mTOR Signalling Pathway

**FIGURE 7:**
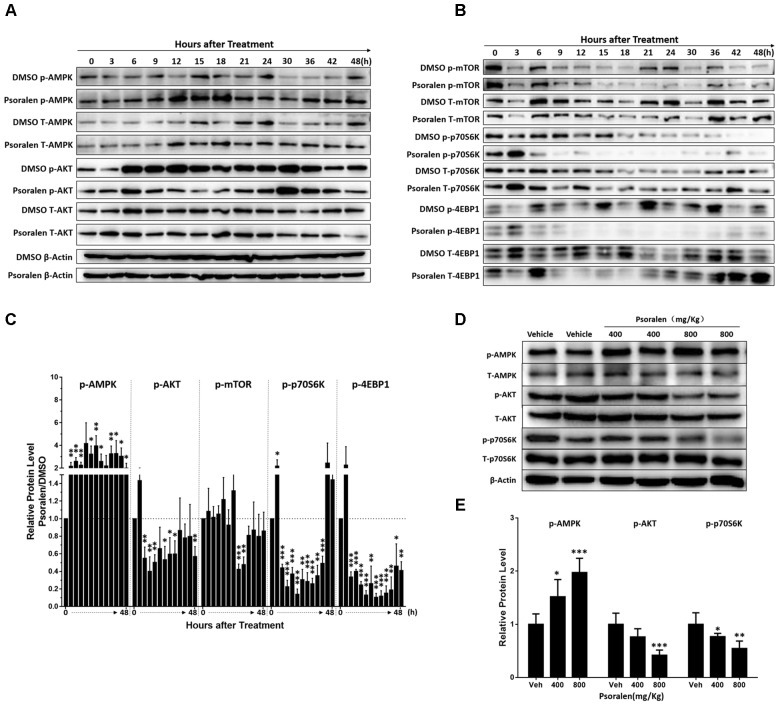
Changes in mTOR signalling pathway-related proteins after psoralen treatment in L02 cells and mice. L02 cells were treated with DMSO or psoralen and incubated for various time periods. When the cells were just beginning to completely adhere (defined as 0 h), the total protein was collected successively every 3 and 6 h during a 48 h period. All experiments were performed three times independently. **(A,B)** Representative immunoblots for the protein levels of total AMPK, AKT, mTOR, p70S6K, and 4EBP1 and their phosphorylated forms. **(C)** The correspondingly relative protein levels were quantified by scanning band intensity and normalised to β-actin. The density of immunoreactive bands was quantified using ImageJ software. Density values were normalised to β-actin and then expressed as the relative expression levels between DMSO-treated and psoralen-treated. C57BL/6 mice were treated with 0.5% CMC-Na solution (vehicle group), or 400 mg/kg or 800 mg/kg psoralen for 24 h. **(D)** Representative immunoblots for the protein levels of total AMPK, AKT, and p70S6K, and their phosphorylated forms. **(E)** The correspondingly relative protein levels were quantified by scanning band intensity and normalised to β-actin (*n* = 6). Data are means ± SDs (*n* = 6). ^∗^*p* < 0.05, ^∗∗^*p* < 0.01, ^∗∗∗^*p* < 0.001 versus DMSO group or vehicle group.

mTOR signalling pathway plays a crucial role in cell proliferation and growth. Thus, the expression levels of various relevant proteins were further analysed, including two major upstream signals, AMPK and AKT, as well as two major downstream signals 4EBP1 and p70S6K. The results showed that L02 cells exposed to psoralen induced a sustained AMPK activation and AKT activation was markedly inhibited at 6–24 h after adhesion (Figures 7A,C). In addition, psoralen treatment significantly inhibited mTOR at 21 and 24 h after adhesion. Although the effects on 4EBP1 and p70S6K were incomplete regarding mTOR, unexpectedly, psoralen caused greater inhibition of 4EBP1 and p70S6K at 6 h after adhesion until 36 or 48 h (Figures 7B,C). These results indicate that psoralen-induced inhibition of mTOR signalling pathway may lead to S-phase arrest in L02 cells. In addition, similar changes in these proteins were detected in the liver of mice. mTOR signalling was inhibited including the activation of AMPK and depression of AKT as well as p70S6K in a dose-dependent manner (Figures 7D,E).

### MHY Alleviated Inhibition of mTOR and S-Phase Arrest Induced by Psoralen *in vitro*

**FIGURE 8:**
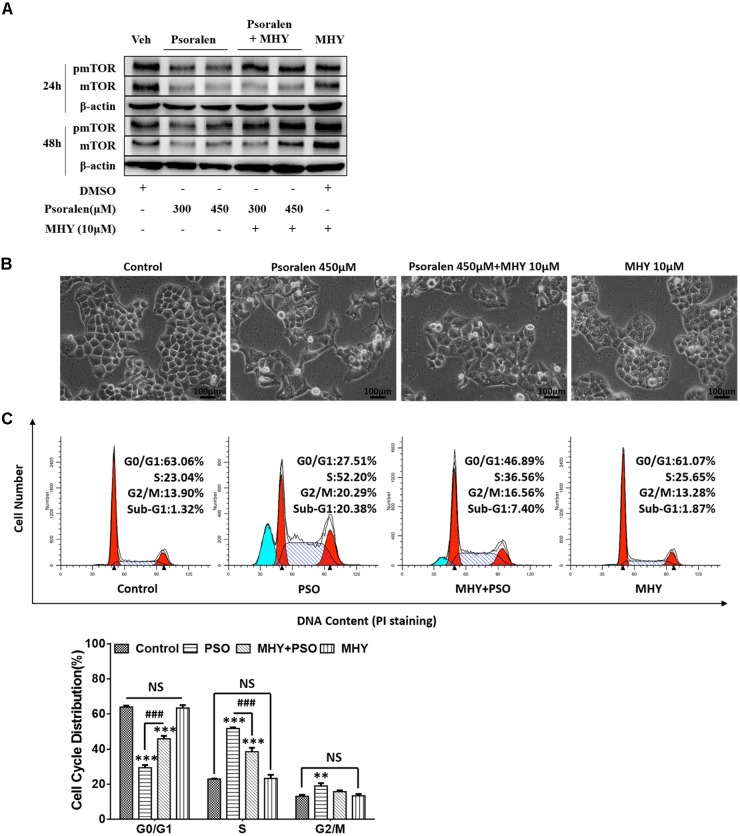
MHY alleviated the inhibition of mTOR and S phase-arrest induced by psoralen in L02 cells. L02 cells were pretreated with 10 μM MHY, an mTOR activator, for 12 h and then co-treated with 300 μM and/or 450 μM psoralen for 24 h and/or 48 h. **(A)** Western blot analysis of mTOR protein levels. **(B)** The representative images of L02 cell morphology. **(C)** Cell cycle analysis using PI staining. Upper panel: representative results of flow cytometry analysis; lower panel: quantitative statistics of cell cycle distribution. PSO, psoralen. All experiments were performed three times independently and the quantitative data are shown as the means ± SDs. ^∗∗^*p* < 0.01, ^∗∗∗^*p* < 0.001 versus control group. ^###^*p* < 0.001, MHY + psoralen group versus psoralen group.

The abovementioned results showed that the mTOR signalling pathway was inhibited both *in vivo* and *in vitro*. Therefore, we determined if MHY, an mTOR activator, could alleviate psoralen-induced S-phase arrest in L02 cells. Western blot analysis showed that MHY could reverse psoralen-induced inhibition of mTOR in a time-dependent manner (Figure 8A), accompanied by the partial recovery of abnormal cell morphology (Figure 8B). In addition, cell cycle analysis showed that psoralen-induced S-phase arrest was significantly attenuated by MHY (Figure 8C). These results showed that mTOR was involved in psoralen-induced S-phase arrest in L02 cells.

### Psoralen-Induced Mitochondrial Damage

**FIGURE 9:**
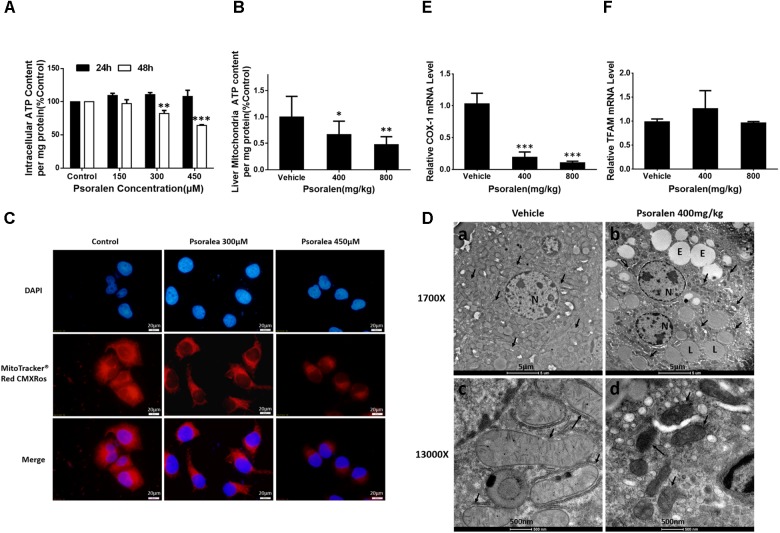
Psoralen induced mitochondrial damage *in vitro* and *in vivo*. L02 cells were treated with DMSO, or 150 μM, 300 μM, or 450 μM psoralen for 24 h or 48 h. **(A)** Intracellular ATP content of L02 cells. **(C)** Intracellular mitochondria of L02 cells were labelled with MitoTracker^®^ Red CMXRos. The experiments were performed three times independently and the quantitative data are shown as the means ± SDs. ^∗∗^*p* < 0.01, ^∗∗∗^*p* < 0.001 versus control group. C57BL/6 mice were treated with 0.5% CMC-Na solution (vehicle group), or 400 mg/kg, or 800 mg/kg psoralen for 24 h. **(B)** ATP content of liver mitochondria in mice. **(D)** Transmission electron microscopy examination of liver sections (*n* = 6). **(a,c)** vehicle group, **(b,d)** psoralen group. Upper panel: original magnification, 1,700×; lower panel: original magnification, 13,000×. Massive mitochondria with normal morphology were observed in the vehicle group and abnormal morphology observed in the psoralen group (black arrow). Hepatocellular oedema (E), lipid droplets (L), and cell nuclei (N) are shown. **(E,F)** Real-time qPCR used to analyse the hepatic mRNA expression levels of COX-1 **(E)** and TFAM **(F)** in mice. Data of **(B,E,F)** are means ± SDs (*n* = 8). ^∗^*p* < 0.05, ^∗∗^*p* < 0.01, ^∗∗∗^*p* < 0.001 versus vehicle group.

AMPK, an intracellular energy sensor, plays a pivotal role in regulating energy and is primarily activated by the increasing ratio of intracellular AMP/ATP or ADP/ATP. ATP production by eukaryotes occurs mainly in the mitochondria and the abovementioned results showed that AMPK was significantly activated by psoralen both *in vivo* and *in vitro*; therefore the ATP content and mitochondrial state were further determined. The results indicated that ATP content was reduced in dose- or concentration-dependent manners after psoralen treatment in both L02 cells (Figure 9A) and liver mitochondria (Figure 9B).

In addition, psoralen significantly decreased mitochondrial density in a concentration-dependent manner, indicated by the decline of red fluorescence (Figure 9C). Moreover, the results of ultrastructural examination using transmission electron microscopy showed numerous mitochondria in the vehicle group (Figure 9D-a) under a low-power field (1700X). Conversely, in the psoralen group, the number of mitochondria markedly decreased with some lipid droplets and oedema in the cytosol (Figure 9D-b). Under a high-power field (13000X), the mitochondrial double-membrane structure and cristae were clearly visible (Figure 9D-c), whereas psoralen treatment led to extremely diminished mitochondria size, almost complete disappearance of cristae, and destruction of membrane integrity (Figure 9D-d), accompanied by declined mRNA levels of mtDNA-encoded cytochrome c oxidase-1 (COX-1), which indicates that mtDNA copy number may have decreased (Figure 9E). However, the mRNA levels of mitochondrial transcription factor A (TFAM) were not significantly changed (Figure 9F). Overall, these results indicate that psoralen can induce mitochondrial damage in both L02 cells and hepatocytes in mice.

## Discussion

In this study, we focused on psoralen to further investigate FP-induced hepatotoxicity and the possible mechanisms involved. Our results indicated that oral administration of 400 mg/kg and 800 mg/kg psoralen in C57BL/6 mice for 24 h caused mild hepatotoxicity (Figure 1).

We performed several *in vitro* experiments to initially explore the possible mechanisms of psoralen-induced hepatotoxicity, and the results showed that psoralen inhibited L02 cell viability mainly by inducing cell cycle arrest at the S phase rather than cell death (Figure 2). The subsequent analyses of molecular mechanisms showed that psoralen-induced S phase arrest was primarily associated with the overexpression of cyclin E1 and p27 (Figures 3A,B). In addition, similar results were found regarding hepatic proteins in the mouse experiments (Figures 3C,D). During cell cycle progression, activation of CDK2–cyclin E complexes drives the cells into the S phase, after which cyclin E is rapidly degraded by ubiquitin-mediated proteolysis (Koepp et al., 2001; Strohmaier et al., 2001). During this phase, cyclin A2 levels gradually increase and associate with CDK2 to form active cyclin A–CDK2 complexes, which maintain progression of the S phase and promote the initiation of DNA synthesis (Hustedt and Durocher, 2017). Therefore, in our study, the overexpression of cyclin E1 occupied the CDK2 binding site and then maybe inhibited the association between cyclin A and CDK2, which induced S-phase arrest. Although currently, there is preliminary consensus on the changes at the molecular level of various cycle-related proteins in the cell cycle progression, controversies remain regarding the precise effects of different cyclin and CDK types. For example, several unexpected compensatory mechanisms were found among cyclins and CDKs (Satyanarayana and Kaldis, 2009). Further studies are necessary to determine more accurate molecular mechanisms.

The liver plays a central role in metabolic homeostasis and detoxification of various metabolites; thus, the liver is highly susceptible to various toxins. The normal liver has remarkable regenerative capabilities allowing continuous performance of its functions despite the injury induced by various toxic chemicals. Based on this knowledge and abovementioned results (Figures 1–3), we speculate that the decrease of liver regenerative and self-healing capabilities induced by hepatocellular cycle arrest play an important role in the hepatotoxicity of psoralen. To verify this hypothesis, we selected the PHx mouse model to evaluate the effect of psoralen on liver regeneration and hepatocellular cycle arrest *in vivo*. In the PHx mouse model, recovery of the lost liver mass and impaired liver function mainly depends on rapid activation from the quiescent state and proliferation of hepatocytes. During the process, hepatocellular cycle arrest induced by any cause is likely to delay liver regeneration (Campbell et al., 2011; Lehmann et al., 2012). Our results indicated that 200 mg/kg psoralen, a dose of almost no hepatotoxicity (Figures 4B–G), significantly delayed liver regeneration as confirmed by the decreased liver regeneration ratio (Figure 5B) and number of proliferative hepatocytes (Figures 5C,D). In addition, the change of cell cycle-related proteins in liver was similar to previous results; psoralen significantly increased cyclin E1 and p27 levels (Figure 6); furthermore, cyclin D1 was inhibited at a specific time point (Figure 6). Overall, these results suggest that hepatocellular cycle arrest was induced by psoralen, which may be responsible for delayed liver regeneration in the PHx mouse model.

200 mg/kg psoralen hardly induced hepatotoxicity in normal mice (Figures 4B–G), but in the PHx mice, acute liver injury induced by hepatectomy was significantly aggravated by psoralen at this dose (Figures 4H–M). These results suggested that psoralen-induced cycle arrest of hepatocytes led to the inhibition of hepatocellular proliferation, which is the predominant event for hepatic recovery from hepatectomy-induced acute liver injury, thereby causing more severe liver injury than in the vehicle group. In normal mice, because the hepatocytes were in a quiescent state, hepatocellular cycle arrest had only a slight effect on liver functions.

Based on the above inference, whether the increased psoralen doses of 400 mg/kg and 800 mg/kg also induced hepatotoxicity in normal mice was uncertain, because the most hepatocytes were in a quiescent state; however, the results indicated that 400 and 800 mg/kg psoralen induced more significant liver injury than 200 mg/kg psoralen in normal mice (Figures 1, 4B–G). Therefore, we inferred that other cycle arrest-independent effects were also involved in the hepatotoxicity induced by higher dose of psoralen, such as cholestasis, due to the mild increased gallbladder/body weight ratio, serum TBA, and TBIL levels (Figures 1A,F–H). Moreover, although the biliary phospholipid and total cholesterol (TC) levels were significantly reduced (Supplementary Figures 4A,B), the concentration of bile acid remained almost unchanged (Supplementary Figure 4C), which indicates that the TBA/lipid ratio increased in the bile. Phospholipids and TC are required for the formation of mixed micelles. Relative increase of bile TBA would lead to increasing the detergent effects of bile acid and damaging cholangiocytes and hepatocytes (De Vree et al., 1998; Jacquemin et al., 2001). This is similar to our previous research, which showed that EtOH extract from FP can induce cholestatic hepatotoxicity (Wang et al., 2012). In addition, our recent research indicated that 8-methoxypsoralen (Methoxsalen), an analogue of psoralen, can induce cholestatic liver injury by disturbing MDR3-mediated phospholipid efflux (Zhao et al., 2017). In this study, meanwhile psoralen still upregulated the proteins levels of cyclin E1 and p27 in normal mice (Figures 3C,D). The results indicated that the effects of cycle arrest in normal mice may be indirectly involved in the hepatotoxicity. For example, hepatocellular cycle arrest enhanced hepatotoxicity induced by cholestasis by attenuating the compensatory capacity of the liver.

Figure 4 showing PHx mice were more sensitive to the hepatotoxicity of psoralen indicate the PHx mouse model may be a more effective or sensitive animal model for evaluating drug-induced liver injury *in vivo*, particularly for drugs with mild hepatotoxicity, such as several herbal and dietary supplements. In addition, liver regeneration plays a vital role in maintaining normal liver function. Thus, if a drugs or chemical inhibit liver regeneration, the risk of liver injury is increased, particularly in people with liver disease. Based on this viewpoint, delayed liver regeneration may be a new indicator for evaluating hepatotoxicity. For example, if a drug could not significantly alter the typical hepatotoxicity indicators, such as those in this study (Figures 4B–G), but could inhibit liver regeneration (Figure 5), it should still be considered hepatotoxic.

After PHx, rapid expression of more than 100 genes not expressed in normal liver is induced (Taub, 1996, 2004) including multiple cytokines, such as HGF, TNF-α, and IL-6. HGF is a direct mitogen for hepatocytes (Michalopoulos, 2007); TNF-α is not a direct mitogen for hepatocytes, but enhances the mitogenic effects of direct mitogens and induces production of IL-6 by activation of NF-κB, which directly upregulates the transcription of *CCND 1/2* (Guttridge et al., 1999; Takeishi et al., 1999). IL-6 binds to its receptor on hepatocytes, activating the STAT3, and then promotes hepatocyte proliferation (Li et al., 2002) or acts as a direct mitogen for biliary cells (Liu et al., 1998). Our results indicated that psoralen dose dependently decreased IL-6, TNF-α, and HGF mRNA expression levels (Figures 5E–G). In addition, immunohistochemistry staining results indicated that the number of inflammatory cells significantly decreased after psoralen treatment in PHx mice (Figures 5C,D). Therefore, the psoralen-induced inhibition of inflammation in the priming stage of liver regeneration was possibly responsible for the subsequent downregulation of cyclin D1, and further induced hepatocellular G1 phase arrest in PHx mice. Thus, unlike psoralen-induced S phase arrest in L02 cells *in vitro*, both G1 and S phases may be simultaneously blocked by psoralen in PHx mice.

Our results showed psoralen inhibited the mTOR signalling pathway *in vitro* and *in vivo*, including mTOR (Figures 7B,C) and its upstream regulators such as AMPK and AKT (Figures 7A,C–E), as well as its downstream effectors such as p70S6K and 4EBP1 (Figures 7B–E). Furthermore, MHY, an mTOR activator, alleviated the effects on S-phase arrest and restored the abnormal morphology induced by psoralen in L02 cells (Figure 8). Overall, these results indicate that inhibition of mTOR signalling was partly responsible for the hepatocellular cycle arrest induced by psoralen. However, the specific interaction between the mTOR signalling pathway and the cycle progression-relevant proteins has not been well described, particularly for cyclin E1 and p27. Therefore, we plan to further investigate the role of the mTOR signalling pathway in psoralen-induced cycle arrest and its relationship with cyclin E1 and p27.

Consistent with AMPK activation, the content of ATP in both L02 cells and mice hepatocellular mitochondria significantly reduced after psoralen treatment (Figures 9A,B). In addition, the results showed that significant mitochondrial damage was induced by psoralen *in vitro* (Figure 9C) and *in vivo* (Figures 9D,E). These results indicate that mitochondrial damage contributed to the hepatotoxicity induced by psoralen, although whether the mitochondrial damage was the cause or result of hepatotoxicity remains unclear in our study and requires further investigation.

There were several clinical reports of liver injury induced by FP and its related preparations; however, the incidence of liver injury was extremely low relative to the widespread use of these drugs. In addition, several researches in China showed that treatment with high-dose FP power or its water extract only induced mild liver injury in rats or mice, which indicated by elevated ALT (about 80–120%) and/or diffuse hepatocellular oedema; it is similar to our results. In this research, the maximal dose of psoralen (800 mg/kg) used for the research of liver injury in mice was almost 65 times the maximum daily intake dose of FP for an adult human stipulated by China Pharmacopoeia (Committee, 2015). To convert the human dose to a mouse dose, the body surface area ratio was used. In general, although our findings and other researches in China indicated that psoralen or FP power or water extract could induce mild liver injury in mice or rats, it was induced at a high dose of these drug after all; moreover, most of the cases of liver injury induced by FP-related preparations had good prognosis after stopping administration and symptomatic treatment. These information indicates that FP is safe or at least low risk of liver injury under standardised use. Whereas, in view of the incidental severe liver injury induced by FP, several FP-related preparations-induced liver injury cases, the mild hepatotoxicity of FP in rodents and our results that psoralen decrease the regenerative capabilities at a dose of almost no hepatotoxicity, it is necessary to be alert to the occurrence of liver injury when using these drugs, especially for the patients with various liver diseases; the hepatotoxicity and decreased liver regenerative capabilities induced by psoralen may increase the risk of severe liver injury in these patients.

## Conclusion

**FIGURE 10:**
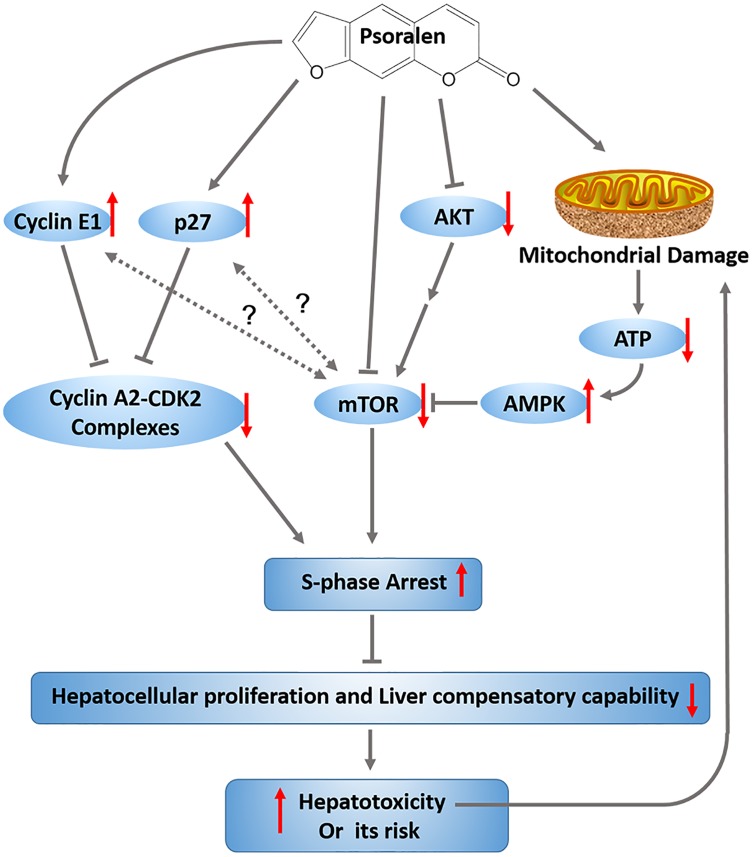
Graphic abstract. The proposed mechanisms involved in psoralen-induced hepatotoxicity.

Our results provide the first evidence that psoralen can induce mild liver injury in mice and cytotoxicity in L02 cells. The investigation of the mechanisms involved indicates that the decrease of liver regenerative and compensatory capabilities induced by hepatocellular cycle arrest may cause the progression of hepatotoxicity associated with the upregulation of cyclin E1 and p27, as well as inhibition of the mTOR signalling pathway and mitochondrial injury (Figure 10).

## Author Contributions

LZ, TW, ZJ, and WZ conceived and designed the experiments. WZ carried out the experiments and wrote the paper. XC, GZ, and DX contributed to the performance of animal experiments. LZ, TW, and ZJ reviewed the manuscript.

## Conflict of Interest Statement

The authors declare that the research was conducted in the absence of any commercial or financial relationships that could be construed as a potential conflict of interest.
