# Supplementary material for: Psoralen Induced Liver Injury by Attenuating Liver Regenerative Capability
Source: Front Pharmacol. 2018 Oct 22;9:1179. doi: 10.3389/fphar.2018.01179 (PMC6232894; doi:10.3389/fphar.2018.01179)
Supplement: Supplementary file 1 [file Data_Sheet_1.PDF]

Supplementary Material

Psoralen Causes Liver Injury by Inducing Hepatocellular Cycle Arrest

Wang Zhou, Xi Chen, Guolin Zhao, Dengqiu Xu, Zhenzhou Jiang, Luyong Zhang*, Tao Wang*

*** Correspondence:** Corresponding Author:

Email address: Luyong Zhang, lyzhang@cpu.edu.cn; Tao Wang, wangtao1331@126.com.
Express delivery address: Jiangsu Key Laboratory of Drug Screening, China Pharmaceutical University, 24 Tong Jia Xiang, Gulou District, Nanjing 210009, China. Fax: + (86) 2583271500, Tel: + (86) 2583271023

1 Supplementary Tables

Supplementary Table 1. The primers used for real-time quantitative PCR in mice

Gene		
IL-6	Forward primer	TACCACTTCACAAGTCGGAGGC
	Reverse primer	CTGCAAGTGCATCATCGTTGTTC
TNF-α	Forward primer	GGTGCCTATGTCTCAGCCTCTT
	Reverse primer	GCCATAGAACTGATGAGAGGGAG
HGF	Forward primer	GATGAGTGTGCCAACAGGTG
	Reverse primer	GTGATGGATACCGTCCCTTT
TFAM	Forward primer	ATGTCTCCGGATCGTTTCAC
	Reverse primer	CCAAAAAGACCTCGTTCAGC
COX-1	Forward primer	GAATGCCACCTTCATCCGAGAAG
	Reverse primer	GCTCACATTGGAGAAGGACTCC
β-Actin	Forward primer	TATTGGCAACGAGCGGTTC
	Reverse primer	ATGCCACAGGATTCCATACCC

Supplementary Table 2. Sequence and results of LD_{50} determination of psoralen in ICR mice

Test sequence	Animal ID	Dose (mg/kg)	Short-term result(48h)	Long-term result(14days)
1	01	350	Survival	Survival
2	02	700	Survival	Survival
3	03	1390	Survival	Survival
4	04	2000	Survival	Survival
5	05	2000	Death	Death
6	06	1390	Survival	Survival
7	07	2000	Death	Death
8	08	1390	Death	Death

ID= identification number; LD_{50}= lethal dose 50

2 Supplementary Figures

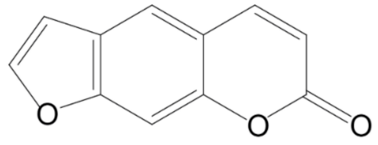

Supplementary Figure 1. The chemical structures of psoralen

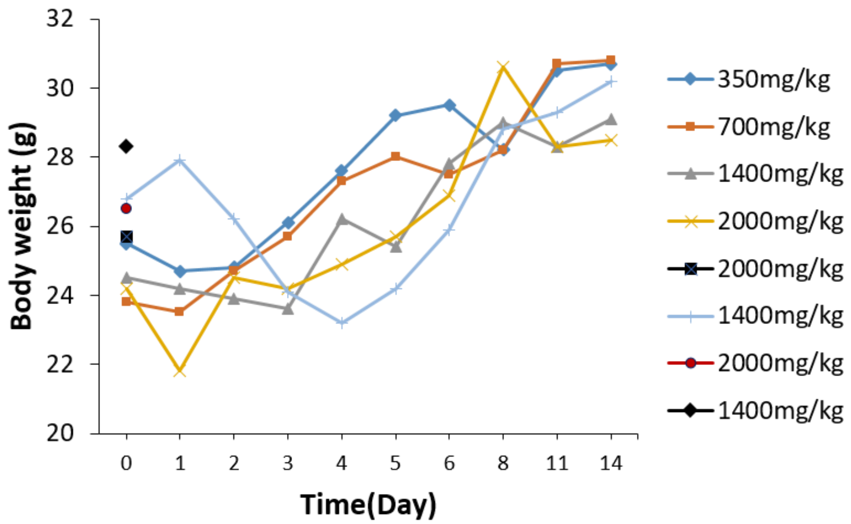

Supplementary Figure 2. Changes in body weight during the determination of acute oral LD50 in ICR mice. 8 ICR mice were treated with psoralen at the dose range of between 350mg/kg and 2000mg/kg within 14 days.

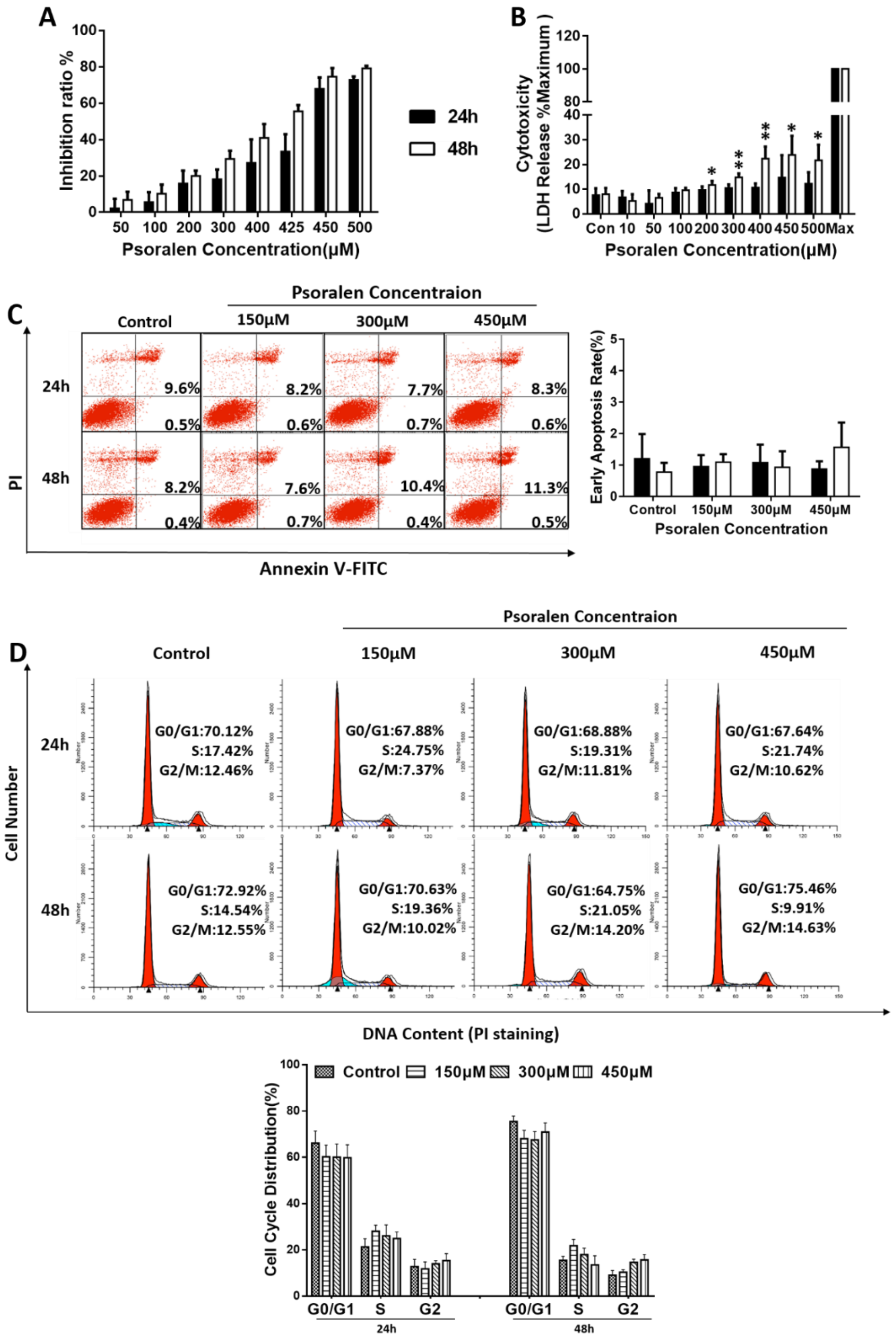

Supplementary Figure 3. Psoralen inhibited the viability of HepG2 cells without apoptosis and cycle arrest. HepG2 cells were treated with DMSO or psoralen. **(A)** MTT assay was used to determine HepG2 cells viability. **(B)** LDH release assay was used to evaluate the cell membrane integrity. **(C)** Apoptotic analysis was performed by annexin V-FITC/PI double staining. Left panel: representative results of flow cytometry analysis; Right panel: quantitative statistics of early apoptotic cells. **(D)** Cell cycle analysis by PI staining. Upper panel: representative results of flow cytometry analysis; Lower panel: quantitative statistics of cell cycle distribution. All experiments were performed three times independently and the quantitative data are shown as the means ± SD. *p<0.05, **p<0.01, ***p<0.001 versus control group.

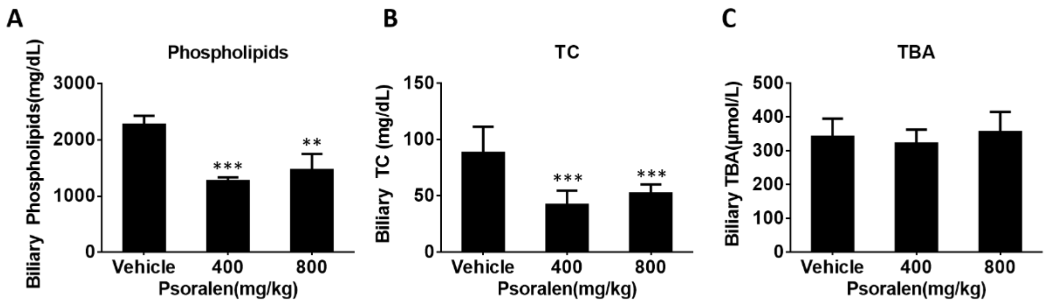

Supplementary Figure 4. Changes in bile composition after psoralen administration. C57BL/6 mice were treated with 0.5% CMC-Na solution (Vehicle group), or psoralen 400mg/kg and 800mg/kg for 24h. **(A)** Bile phospholipids were determined using Phospholipids C test (Wako Pure Chemicals, Osaka, Japan). **(B)** Bile Total Cholesterol (TC) was detected using a commercial kit according to the manufacturer's protocol (Whitman Biotech, Nanjing, China). **(C)** Bile TBA was determined using an HITACHI7080 Automatic Clinical Analyzer (HITACHI, Japan). Data are means ±SD (n=8). *p<0.05, **p<0.01, ***p<0.001 versus vehicle group.

3 Abbreviations

4E-BP1: eukaryotic translation initiation factor 4E (eIF4E) binding protein 1
ADP: adenosine diphosphate
AKT: protein kinase B
AMP: adenosine monophosphate
AMPK: adenosine monophosphate-activated protein kinase
ATP: adenosine triphosphate
CDK: cyclin-dependent kinase
CMC-Na: sodium carboxymethylcellulose
DAPI: 4',6-diamidino-2-phenylindole
DMSO: Dimethyl sulfoxide
HGF: hepatocyte growth factor
HPLC: High-performance liquid chromatography
IL-6: interleukin-6
MDR3: multidrug resistance 3 P glycoprotein
mTORC1: mammalian target of rapamycin complex 1
NF-κB: nuclear factor-kappa B
p70S6K:70 kDa ribosomal protein S6 kinase
PCNA: proliferating cell nuclear antigen
STAT3: signal transducer and activator of transcription 3
TNF-α: tumor necrosis factor alpha
